# Polycaprolactone nanofiber scaffold enhances the osteogenic differentiation potency of various human tissue-derived mesenchymal stem cells

**DOI:** 10.1186/s13287-017-0588-0

**Published:** 2017-06-24

**Authors:** Ruyue Xue, Yuna Qian, Linhao Li, Guidong Yao, Li Yang, Yingpu Sun

**Affiliations:** 1 Reproductive Medical Center, First Affiliated Hospital of Zhengzhou University, Zhengzhou, 450052 China; 2 Key Laboratory of Biorheological Science and Technology, Ministry of Education, Bioengineering College, Chongqing University, Chongqing, 400044 China; 3 Key Laboratory for Biomechanics and Mechanobiology of Ministry of Education, School of Biological Science and Medical Engineering, Beihang University, Beijing, 100191 China

**Keywords:** Polycaprolactone, Mesenchymal stem cells, Osteogenic differentiation, Wnt/β-catenin signaling pathway, Smad3

## Abstract

**Background:**

Polycaprolactone (PCL) has been regarded as a promising synthetic material for bone tissue engineering application. Owing to its unique biochemical properties and great compatibility, PCL fibers have come to be explored as a potential delivering scaffold for stem cells to support bone regeneration during clinical application.

**Methods:**

The human derived mesenchymal stem cells (MSCs) were obtained from umbilical cord (UC), bone marrow (BM), and adipose tissue (AD), respectively. The osteogenic differentiation potency of various human MSCs on this novel synthetic biomaterial was also investigated in vitro.

**Results:**

Here, we illustrated that those human UC-, BM-, and AD-derived MSCs exhibited fibroblast-like morphology and expressed characteristic markers. Impressively, PCL nanofiber scaffold could support those MSC adhesion and proliferation. Long-term culture on PCL nanofiber scaffold maintained the viability as well as accelerated the proliferation of those three different kinds of human MSCs. More importantly, the osteogenic differentiation potency of those human MSCs was increased significantly by culturing on PCL nanofiber scaffold. Of note, BM-derived MSCs demonstrated greater differentiation potency among the three kinds of MSCs. The Wnt/β-catenin and Smad3 signaling pathways contributed to the enhanced osteogenesis of human MSCs, which was activated consistently by PCL nanofiber scaffold.

**Conclusions:**

The utilization of PCL nanofiber scaffold would provide a great application potential for MSC-based bone tissue repair by enhancing the osteogenic differentiation of human MSCs.

## Background

Mesenchymal stem cells (MSCs) are a kind of cell population with multi-differentiation potential, which were first identified in bone marrow (BM)-MSCs more than 40 years ago [1]. It was soon noted that MSCs display not only regenerative properties, but also remarkable differentiation ability. Growing evidence showed that human MSCs are able to differentiate into derivatives of all germ layers including bone, cartilage, adipose tissue, cardiomyocyte, and neurocyte [2, 3]. Those properties have endowed MSC-based therapy with a great potential therapeutic strategy for wound healing and tissue regeneration, which is also thanks to few ethical issues and low risk of tumorigenesis in contrast to embryonic stem cells and induced pluripotent stem cells [4–6]. Even though bone marrow is referred to as the most extensively used source for MSC isolation, the frequency of BM-MSCs has been estimated to be in the order of 0.001–0.01% of total uncleated cells in bone marrow [7, 8]. Instead, several other tissue-derived MSCs have been identified and investigated. Among them, umbilical cord (UC)-MSCs and adipose tissue (AD)-MSCs exert great potential as ideal allergenic and autologous cell types for clinical application owing to their easy cell isolation and sufficient frequency of clean sources [9–11]. Increasing studies have indicated that BM-MSCs, UC-MSCs, and AD-MSCs are able to differentiate into bone tissues in vitro and in vivo. But there still exist concerns regarding the osteogenic efficiency of those most widely used types of MSCs during clinical application.

To address this concern, various synthetic biomaterials have been employed to support the adhesion and accelerate the osteogenic differentiation in MSC-based regenerative therapy for treating bone impairment. Notably, the use of polycaprolactone (PCL) for tissue engineering increased significantly over the past decade [12–14]. PCL, a biodegradable polyester material, has been widely exploited in several kinds of implants, adhesion barriers, and drug delivery devices. Since the 1980s, this material has been approved by the Food and Drug Administration (FDA), which is thanks to its excellent electrospinnability, good mechanical features, good compatibility, and biodegradable properties. But the effects of this biomaterial on the osteogenic potency of various human MSCs were still elusive.

Here we used a novel scaffold consisting of PCL-based electrospun nanofibers and investigated the bioactivity and osteogenic potency of three different tissue-derived human MSCs on this synthetic biomaterial. It was illustrated that human-derived MSCs obtained from umbilical cord, bone marrow, and adipose tissue all could form colonies, show fibroblast-like morphology, and be characterized by expressing CD90, CD105, and CD73 but not CD31, CD34, and CD45. Long-term culture on this novel synthetic scaffold did not reduce human MSC viability. Moreover, the proliferation potency of human UC-MSCs, BM-MSCs, and AD-MSCs was increased after adhesion on the surface of the PCL nanofiber scaffold. We further investigated the osteogenic differentiation of human UC-MSCs, BM-MSCs, and AD-MSCs respectively with and without PCL nanofiber scaffold, then we found that this synthetic scaffold indeed enhanced the osteogenic potency of those three different tissue-derived human MSCs in vitro. In addition, human BM-MSCs exert the greatest increase of osteogenic potency among those three kinds of human MSCs when cultured on PCL nanofiber scaffold. The Wnt/β-catenin and Smad3 signaling pathways contributed to the osteogenesis of human MSCs, which was activated consistently by the PCL nanofiber scaffold. Therefore, our study indicated that PCL nanofiber scaffold exerts a great compatibility with human MSCs, which could maintain cell viability as well as promote the proliferation potency. More importantly, the osteogenic potency of human UC-MSCs, BM-MSCs, and AD-MSCs could be enhanced by culturing on PCL nanofiber scaffold, implying the great potential of this novel synthetic biomaterial combined with MSC-based therapy for bone repair in clinical application.

## Methods

### Polycaprolactone (PCL)

PCL is a kind of polymer with mechanical properties, miscibility and biodegradability. PCL is an aliphatic linear polyester, with a glass transition temperature of about −60 °C and a melting point of 55–60 °C, depending on the degree of crystallinity, which in turn is dictated by the molecular weight and the scaffold fabrication process. It is also a kind of biocompatible, absorbable and low-cost synthetic polymer. The way to obtain high molecular weight PCL is mainly dependent of the ring-opening polymerization of ε-caprolactone (Fig. 2a). Due to its semi-crystalline and hydrophobic nature, the mechanical property of PCL is suitable for a variety of applications, and this material demonstrates a slow degradation rate (2–4 years). PCL has been clinically used as a slow-release drug delivery device and suture material that has been approved by the FDA since the 1980s. In this study, the PCL nanofiber scaffold was fabricated by the Key Laboratory of Biorheological Science and Technology, Ministry of Education, Bioengineering College, Chongqing University.

### Culture of human-derived MSCs

UC-MSCs (c-12971), BM-MSCs (c-12974), and AD-MSCs (c-12977) were purchased from Promocell (Miaotong (Shanghai) Biological Science & Technology Co., Ltd. Shanghai, China). Cells were cultured with Dulbecco’s modified Eagle’s medium (DMEM) supplemented with 10% FBS, 10 ng/mL bFGF and 5% penicillin/streptomycin and incubated at 37 °C and 5% CO_2_. As shown in the manufacturer’s instructions, the three kinds of tissue were obtained from healthy volunteers after informed consent according to the Helsinki declaration.

### MSC surface marker identification

The MSCs surface markers were examined by flow cytometry using PE-conjugated anti-human CD45, CD34, CD31, CD73, CD90, and CD105 antibodies were used for staining human mesenchymal stem cells according to the manufacturer’s instructions. All antibodies were purchased from BD Biosciences (Franklin Lakes, NJ, USA). Briefly, 1 × 10^5^ MSCs were harvested with 0.25% Trypsin/EDTA, washed with PBS twice, and then incubated with monoclonal antibody in 100 μL PBS at 4 °C for 30 minutes. Then the cells were washed in PBS three times and resuspended in a volume of 300 μL PBS. Fluorescence of the cells was detected by flow cytometer (FACS Caliber, BD).

### Cell viability detection

MSC viability was examined by flow cytometry analysis using LIVE/DEAD® Fixable Dead Cell Stain Kit, which was purchased from Life Technologies (Thermo Fisher Scientific, Waltham, MA, USA). Briefly, 1 × 10^5^ MSCs were harvested with 0.25% Trypsin/EDTA, washed with PBS twice, and then incubated with 1 μL of DMSO-diluted stain (Cat. No. L23101) in 1 mL protein-free buffer at 4 °C for 30 minutes. Then the cells were washed in PBS three times and resuspended in a volume of 300 μL PBS. Fluorescence of the cells was detected by flow cytometer (FACS Caliber, BD).

### Cell proliferation assay

1 × 10^4^ MSCs were seeded in a six-well plate and then the cells were continuously cultured for a further 14 days. Each of the three wells of MSCs were harvested with 0.25% Trypsin/EDTA, washed with PBS twice, and then counted by cell counting counter (Countstar, Shanghai Ruiyu BioTech Co., Ltd., Shanghai, China) every 2 days. The proliferation curve was made by using GraphPad Prism (v 6.0, GraphPad Software, Inc., San Diego, CA, USA).

### Osteogenic differentiation of MSCs

MSCs were seeded in 24-well culture plates in a density of 2 × 10^4^ cells for each well with osteogenic culture medium to induce osteogenic differentiation for 21 days. The medium was refreshed every 2 days. Inducing osteogenic differentiation medium contained 10% FBS, 100 μg/mL streptomycin, 100 U/ml penicillin, 10 mM β-glycerolphosphate, 0.1 μM dexamethasone, and 0.2 mM ascorbate. At the 21th day of differentiation, the MSCs were fixed with 4% formaldehyde and Alizarin Red S staining was employed to examine the osteogenic differentiation of MSCs.

### Real-time PCR

Trizol reagent (Invitrogen, Carlsbad, CA, USA) was used to isolate total RNA according to the introductions from the manufacturer. RevertAid RT-PCR system (Fermentas, Waltham, MA, USA) was employed to reverse-transcribe the total RNA into cDNA. Then the cDNA was mixed with primers and Maxima SYBR Green qPCR Master Mix in the Real-time PCR Stratagene Mx3000P System (Applied Biosystems, Foster City, CA, USA). The levels of mRNA in each group were compared after normalization by GAPDH. The primer sequences are listed in Table 1.

**Table 1:** Sequence of the oligonucleotides for real-time PCR

Gene	Sequence (5′ → 3′)	
β-actin	F	GTGGGGCGCCCCAGGCACCA
	R	CTTCCTTAATGTCACGCACGATTTC
Runx-2	F	ACGACAACCGCACCATGGT
	R	CTGTAATCTGACTCTGTCCT
ALP	F	TGGAGCTTCAGAAGCTCAACACCA
	R	ATCTCGTTGTCTGAGTACCAGTCC
Collagen I	F	CCTGAGCCAGCAGATTGA
	R	TCCGCTCTTCCAGTCAG
BMP-2	F	GAGGTCCTGAGCGAGTTCGA
	R	ACCTGAGTGCCTGCGATACA

### Western blotting analysis

Total proteins extraction from MSCs and Western blot analysis were performed as described [15]. Briefly, MSCs were lysed in RIPA buffer with 1 mM PMSF at 0 °C. The cell lysates were centrifuged in 4 °C at 12,000 rpm for 10 minutes. Then the protein supernatants were transferred into new tubes. The concentration of protein in each sample was assessed by BCA Protein Assay Kit. The protein was mixed with laemmli sample buffer, heated at for 10 minutes at 65 °C. The samples were loaded (20 μg per sample) and separated by sodium dodecyl sulfate-polyacrylamide gel (7.5%) electrophoresis in denaturing conditions and electroblotted on nitrocellulose membranes. The membranes were blocked by Tris-buffered saline containing Tween 20 (TBST) with 5% nonfat milk at room temperature for 2 hours. Primary antibodies of β-catenin (610154, BD Biosciences), Smad3 (ab40854, Abcam, Cambridge, MA, USA), and p-Smad3 (ab52903, Abcam) were incubated with the membranes at 4 °C overnight. Then the membranes were incubated with horseradish peroxidase (HRP)-conjugated secondary antibodies (Dako, Glostrup, Denmark) and the results were observed by enhanced chemiluminescence. GAPDH was employed as internal control to normalize the loading protein.

### Scanning electron microscope

The scanning electron microscope (SEM; JEOL 5300, JEOL USA Inc., Peabody, MA, USA) was used to observe the MSCs attachment on PLC. Specimens with MSCs were rinsed with PBS buffer and fixed with 2.5% (v/v) glutaraldehyde in a 0.1 mol/L sodium cacodylate buffer for 2 hours and then were post fixed in 1% (w/v) OsO4 for 1 hour. The specimens were subjected to graded alcohol dehydration, washed with hexamethyldisilazane, coated with gold, and observed by SEM.

### Statistical analysis

All data, expressed as mean ± standard deviation (SD), were from at least three separate experiments. Statistical analysis was performed by *t* test (two-tailed) by SPSS 18.0 software (SPSS, Inc., Chicago, IL, USA). *P* < 0.05 was considered to be statistically significant. All experiments were performed at least in triplicate.

## Results

### Isolation and identification of human MSCs

**Fig. 1:**
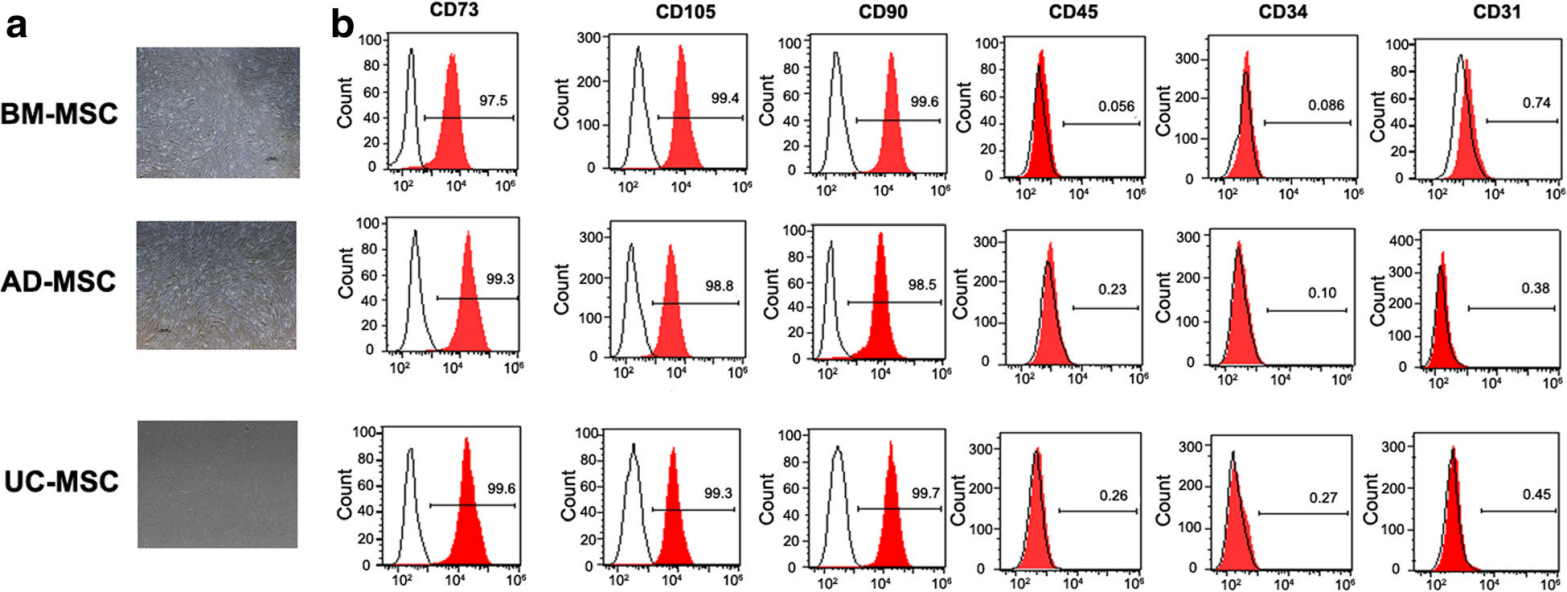
Identification of human MSCs derived from adipose tissue, bone marrow, and umbilical cord. **a** The cell morphology was investigated under scanning electron microscope. **b** Human MSCs were identified by flow cytometry analysis on the expression of specific surface markers such as CD73, CD105, CD90, CD45, CD34, and CD31. *AD-MSC* adipose tissue-derived MSCs, *BM-MSC* human bone marrow-derived MSCs, *UC-MSC* umbilical cord-derived MSCs

Human MSCs obtained from umbilical cord, bone marrow and adipose tissue adhered to the plastic surface, showed fibroblast-like morphology, and began to form colonies under microscope investigation (Fig. 1a). Then the surface markers of MSCs were identified by flow cytometry analysis. The results demonstrated that the three different tissue-derived MSCs were positive for the surface markers such as CD105, CD73, and CD90, and negative for surface markers including CD45, CD31, and CD34 (Fig. 1b).

### PCL nanofiber scaffold increased the cellular proliferation of human MSCs

**Fig. 2:**
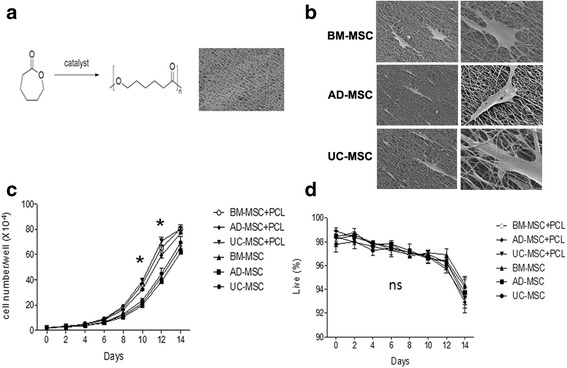
Proliferation and viability of human MSCs grown on PCL material. **a** The structure of PCL nanofiber scaffolds. **b** Human MSCs were cultured on PCL nanofiber scaffolds with an enlarged morphology under electron microscope. **c** Cell proliferation of human MSCs on PCL nanofiber scaffolds was detected, ^*^ *P* < 0.05. **d** Cell viability of human MSCs on PCL nanofiber scaffolds was detected. *AD-MSC* adipose tissue-derived MSCs, *BM-MSC* human bone marrow-derived MSCs, *ns* not significant, *PCL* polycaprolactone, *UC-MSC* umbilical cord-derived MSCs

PCL nanofiber scaffolds were fabricated by using fused deposition modeling FDM (Fig. 2a). The deposited fibers were ellipsoidal in their cross section, which contain semiaxes of 170 and 120 μm, respectively. The thickness of each layer was thus 120 μm. The distance of center-center fiber in each deposited layer was 1.0 mm, and the fiber orientation of each consecutive layer was shifted 0.17 mm and angled at 105°. In addition, the analysis by SEM demonstrated that the scaffolds were completely coated with a hydroxyapatite/tricalcium phosphate (HA/TCP) layer on the PCL fibers and in the pores between the fibers [16]. The analysis and mapping of the element components by energy dispersive X-ray (EDX) showed that the TCP was dispersed on the scaffold-coating layer uniformly (Fig. 2a). To detect the effect of PCL nanofiber scaffold on the function of human MSCs, human UC-, BM-, and AD-derived MSCs were cultured on PCL nanofiber scaffolds. Impressively, PCL nanofiber scaffold was capable of supporting those MSCs adhesion and proliferation (Fig. 2b). Long-term culture on PCL nanofiber scaffolds maintained the viability as well as accelerated the proliferation of the three different kinds of human MSCs (Fig. 2c, d).

### The osteogenic differentiation was promoted in human UC-MSCs, BM-MSCs, and AD-MSCs within the PCL nanofiber scaffold

**Fig. 3:**
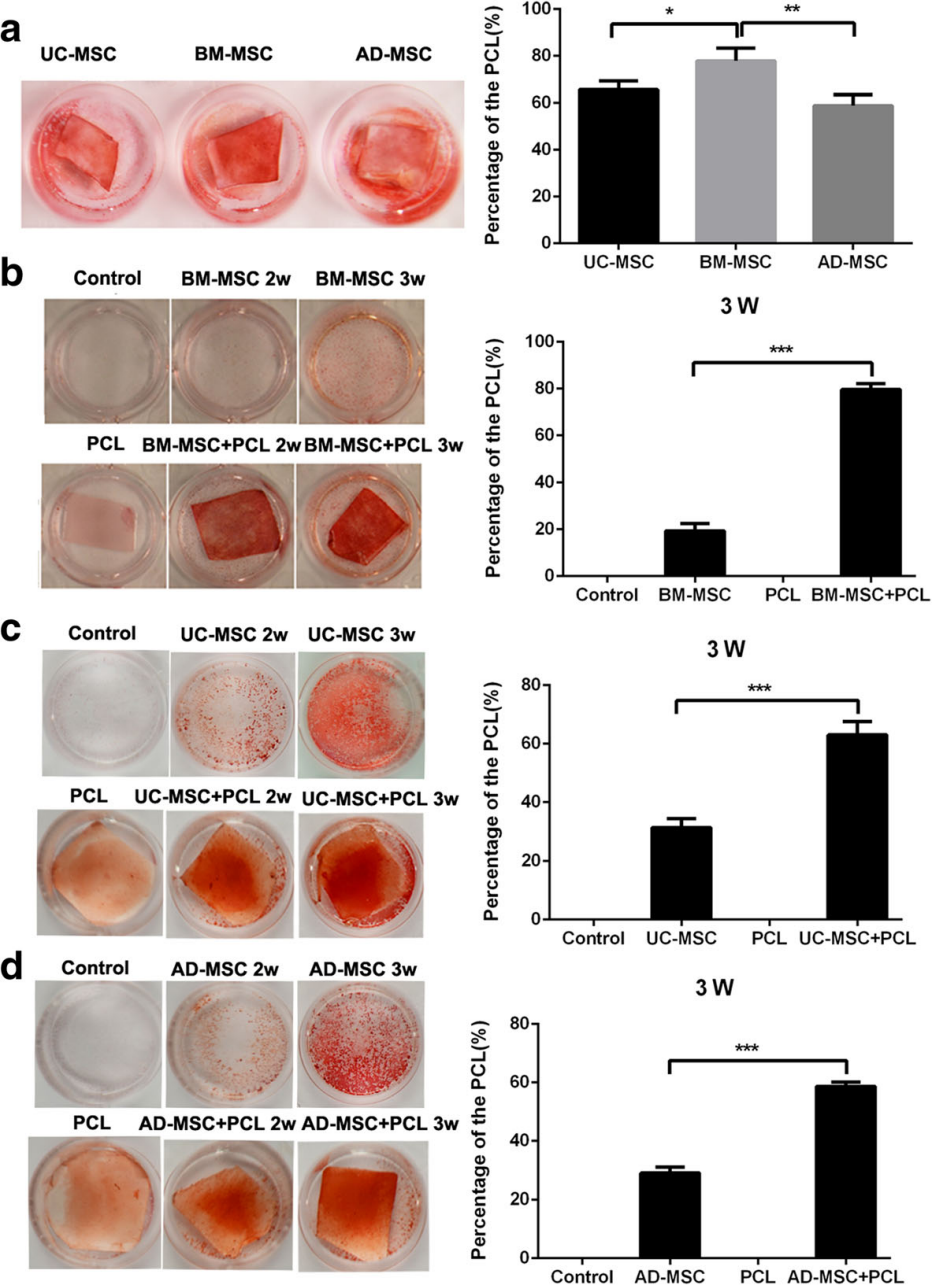
Comparison of the osteogenic differentiation potency among three kinds of human MSCs grown on PCL material. **a** Evaluation of the osteogenic differentiation potency among three kinds of human MSCs cultured on PCL material, ^*^ *P* < 0.05, ^**^ *P* < 0.01. **b**–**d** Comparison of the osteogenic differentiation potency of three kinds of human MSCs cultured on PCL material or normal culture dish respectively. ^***^ *P* < 0.001. *AD-MSC* adipose tissue-derived MSCs, *BM-MSC* human bone marrow-derived MSCs, *PCL* polycaprolactone, *UC-MSC* umbilical cord-derived MSCs

**Fig. 4:**
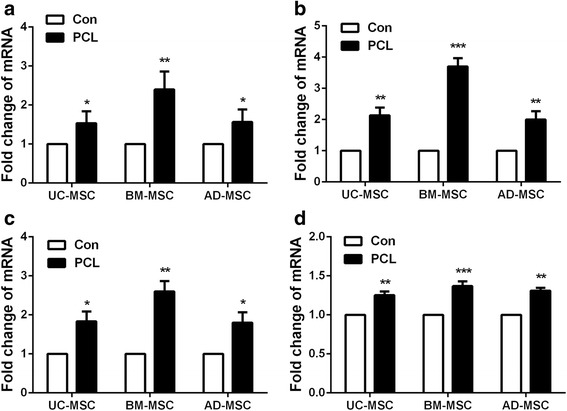
The expression of osteo-specific genes *ALP*, *BMP-2*, *RUNX2*, and *COLL1A1* among three kinds of human MSCs. **a**–**d** Real-time PCR was employed to examine the expression of osteo-specific genes *ALP*, *BMP-2*, *RUNX2*, and *COLL1A1* in UC-MSC, BM-MSC, and AD-MSC on PCL nanofiber scaffolds. ^*^ *P* < 0.05; ^**^ *P* < 0.01; ^***^ *P* < 0.001. *AD-MSC* adipose tissue-derived MSCs, *BM-MSC* human bone marrow-derived MSCs, *UC-MSC* umbilical cord-derived MSCs

Alizarin Red S staining was employed to observe the calcium deposition in the osteogenic differentiation of MSCs on PCL scaffolds at 21 days post-differentiation. Alizarin Red-positive nodules formed in UC-MSCs, BM-MSCs, and AD-MSCs on PCL scaffolds uniformly. And a slightly higher maximal ability of osteogenic formation was present in BM-MSCs compared to UC-MSCs and AD-MSCs on scaffolds (Fig. 3a). Then the osteogenic differentiation of three tissue-derived human MSCs on PCL scaffolds was detected at 2 and 3 weeks. The results showed that PCL nanofiber scaffold could enhance the osteogenic differentiation of the three kinds of MSCs (Fig. 3b–d), among which, BM-MSCs showed more significant changes. To further investigate the effects of PCL nanofiber scaffold on osteogenic differentiation of the three tissue-derived MSCs, the gene expression of osteogenic markers *ALP, BMP-2, RUNX2,* and *COLL1A1* were determined by RT-PCR. Results obtained from RT-PCR were quantitative and presented as a ‘fold change’ in mRNA levels in MSCs on PCL when compared to hMSCs on normal scaffolds (Fig. 4a–d), indicating that PCL scaffold promoted the osteogenic differentiation of MSCs.

### Wnt/β-catenin and Smad3 signaling pathways were activated in human MSCs within the PCL nanofiber scaffold

**Fig. 5:**
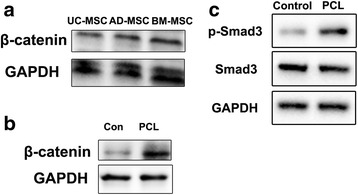
Wnt/β-catenin and Smad3 signaling pathways were activated in human MSCs grown on PCL nanofiber scaffolds. **a** Western blotting was used to detect the expression of β-catenin in UC-MSCs, BM-MSCs, and AD-MSCs. **b**, **c** Western blotting was used to detect the expression of β-catenin, Smad3, and p-Smad3 in BM-MSCs on PCL nanofiber scaffolds. *AD-MSC* adipose tissue-derived MSCs, *BM-MSC* human bone marrow-derived MSCs, *PCL* polycaprolactone, *UC-MSC* umbilical cord-derived MSCs

To confirm the above findings, suggesting that Wnt/β-catenin signaling is involved in the observed phenomena, the expression of β-catenin was determined by Western blot analysis. The results demonstrated higher expression of β-catenin in the human BM-MSCs on PCL nanofiber scaffolds than control (Fig. 5a). Consistently, the Wnt/β-catenin signaling pathway was also upregulated in UC-MSCs and AD-MSCs on scaffolds, but compared with those two groups, the level of β-catenin in the human BM-MSCs on PCL nanofiber scaffolds was significantly increased (Fig. 5a and b). Besides that, the expression of Smad3 and p-Smad3 in the human BM-MSCs cultured on PCL nanofiber scaffolds was also attested, showing that the nano-scaffold did not lead to upregulation of Smad3, but effectively upregulated the expression of p-Smad3 (Fig. 5c). These results indicated that PCL nanofiber scaffolds could enhance the osteogenic differentiation of human MSCs through both the Wnt/β-catenin and Smad3 signaling pathway.

**Fig. 6:**
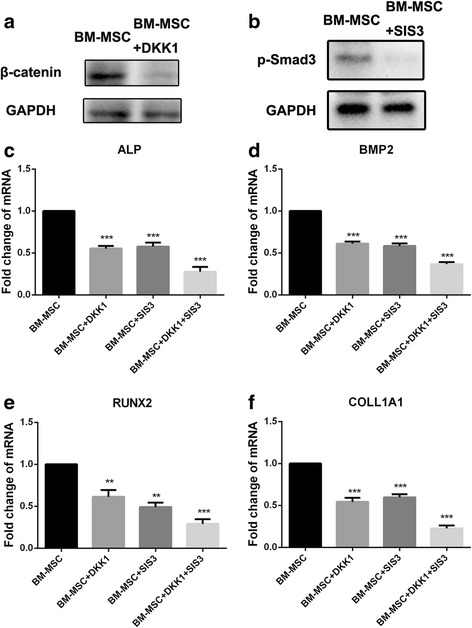
The effect of Wnt/β-catenin signaling inhibitor (DKK1) and Smad3 inhibitor (SIS3) on the expression of osteo-specific genes in BM-MSCs on PCL nanofiber scaffolds. **a** Western blotting was used to detect the expression of β-catenin in DKK1-treated BM-MSCs on PCL nanofiber scaffolds. **b** Western blotting was used to detect the activation of Smad3 in SIS3-treated BM-MSC on PCL nanofiber scaffolds. **c**-**f** Real-time PCR was performed to examine the expression of osteo-specific genes in DKK1 and/or SIS3-treated BM-MSCs on PCL nanofiber scaffolds. ^*^ *P* < 0.05; ^**^ *P* < 0.01; ^***^ *P* < 0.001. *BM-MSC* human bone marrow-derived MSCs, *DKK1* Wnt/β-catenin signaling inhibitor, *SIS3* Smad3 signaling inhibitor

To verify the involvement of the Wnt/β-catenin and Smad3 signaling pathways, we evaluated the inhibitory effect of these pathways on osteogenesis in hMSCs on PCL nanofiber scaffolds. First, Wnt/β-catenin signaling inhibitor (DKK1) and Smad3 inhibitor (SIS3) both could effectively inhibit the expression of β-catenin and p-Smad3 respectively in MSCs on PCL nanofiber scaffolds (Fig. 6a and b). Then, the expression of the osteo-specific genes *ALP, BMP-2, RUNX2*, and *COLL1A1* were examined in the inhibitor-treated BM-MSCs on the PCL nanofiber scaffolds. The results showed that single inhibitors could lead to the downregulation of osteo-specific genes in BM-MSCs on PCL nanofiber scaffolds compared with the control group. However, the combination of DKK1 and SIS3 could effectively enhance the downregulation of osteo-specific genes compared with single treated groups (Fig. 6c–f). These results indicated that the Wnt/β-catenin signaling pathway and Smad3-associated signaling pathway could be activated by PCL nanofiber scaffolds in BM-MSCs, which enhance the osteogenic differentiation of MSCs.

## Discussion

The present in vitro study was carried out to evaluate the changes in osteogenesis levels of human MSCs placed on PCL nanofiber scaffolds [12]. PCL is a biodegradable polyester material, which is widely used in several kinds of implants, adhesion barriers, and drug delivery devices that have been approved by the FDA. More recently, PCL has also been exploited as a synthetic polymer in electrospun fiber fabrication depending on its favorable mechanical properties, remarkable electrospinnability, good blend compatibility, and it also shows a degradable feature [17, 18]. To establish an ideal microenvironment for cellular responses and elicit specific osteochondral formation, the surface structure of this scaffold is supposed to emulate one or more of the osteochondral-forming components [19]. So PCL fibers have come to be explored as a potential delivering scaffold for stem cells to support bone regeneration.

Bone is considered as a dynamic tissue which has an innate propensity to repair injury without scarring [20]. MSC differentiation into osteoblasts plays a crucial role in bone regeneration and remodeling during the whole life. MSCs also have multi-potential to differentiate into osteogenic, adipogenic, and chondrogenic lineages [2, 21]. Bone marrow-derived MSCs are regarded as a suitable cell source for tissue engineering owing to their great osteogenic capability [19]. In this study, the human MSCs were isolated from UC, BM, and AD tissues, respectively. The osteogenic differentiation potency of various human MSCs on PCL nanofiber scaffold was investigated in vitro. We found that the osteogenic differentiation potency of those human MSCs was increased significantly by culturing on PCL nanofiber scaffolds, and BM-derived MSCs demonstrated greater differentiation potency among the three kinds of MSCs.

The canonical Wnt and Smad-dependent BMP pathways interact together and play important roles in osteogenesis [20, 22–26]. However, the crosstalk between those signaling pathways during MSC-mediated regeneration remains elusive. In our study, the Wnt/β-catenin and Smad3 signaling pathways were activated in the osteogenesis of human MSCs on PCL nanofiber scaffolds. The Wnt signal is able to modulate several developmental processes, including cell adhesion, gene expression, and tissue homeostasis [27–29]. Additionally, the Wnt/β-catenin signaling pathway plays a crucial role in MSC growth and differentiation [30–32]. The results from human and mouse genetics analysis have shown that Wnt/β-catenin signaling contributes to the modulation of bone formation [33]. And Smad3 could induce the expression of the genes related to the osteoblast phenotype [34]. Therefore, the activation of Wnt/β-catenin and Smad3-mediated signaling is a useful marker for stem cell growth and osteogenic differentiation.

## Conclusions

In summary, we found that PCL nanofiber scaffolds exert a great effect on maintaining human MSCs viability as well as promoting their proliferation potency. More importantly, the osteogenic potency of human MSCs was enhanced by culturing on PCL nanofiber scaffold, implying the great potential of this novel synthetic biomaterial combined with MSC-based therapy for bone repair in clinical application. Also, the activation of Wnt/β-catenin signaling and Smad3-associated signaling pathway contributes to this enhanced osteogenic differentiation of human MSCs mediated by PCL nanofiber scaffolds.

## Abbreviations

AD: Adipose tissue
BM: Bone marrow
DMEM: Dulbecco’s modified Eagle’s medium
FDA: Food and Drug Administration
HRP: Horseradish peroxidase
MSCs: Mesenchymal stem cells
PCL: Polycaprolactone
SEM: Scanning electron microscope
UC: Umbilical cord
